# Pembrolizumab-induced oral erosive lichen planus treated with dupilumab

**DOI:** 10.1016/j.jdcr.2026.01.027

**Published:** 2026-01-29

**Authors:** Lisa S. Mun, Ndidi Enwereji, Steven Daveluy

**Affiliations:** a Central Michigan University College of Medicine, Saginaw, Michigan; b Department of Dermatology, Wayne State University School of Medicine, Detroit, Michigan

**Keywords:** dupilumab, immune checkpoint inhibitors, immune-related cutaneous adverse events, oral erosive lichen planus, pembrolizumab

## Introduction

Pembrolizumab is an Federal Drug Administration-approved humanized monoclonal antibody targeting programmed cell death protein 1 widely used in cancer immunotherapy. It belongs to the class of immune checkpoint inhibitors (ICIs), which have demonstrated substantial efficacy in treating various malignancies. However, ICIs can lead to widespread immune activation, resulting in immune-related adverse events, including immune-related cutaneous adverse events (ircAEs), which occur in up to 30% to 60% of patients.^1^, ^2^, ^3^ Common ircAEs include eczematous, morbilliform, bullous, and lichenoid eruptions, vitiligo, and pruritus.^1^, ^2^, ^3^, ^4^, ^5^ Management typically involves topical corticosteroids for mild to moderate cases,^2^^,^^3^ while systemic corticosteroids and immunomodulators such as dupilumab are considered for more severe or steroid-refractory presentations.^4^^,^^6^ We report a case of pembrolizumab-induced oral erosive lichen planus (LP) successfully managed with dupilumab.

## Case presentation

A 59-year-old woman with recurrent oral cavity squamous cell carcinoma presented with a 3-week history of painful oral erosions, a pruritic morbilliform eruption, and a nonhealing forearm graft site ulcer. Her oncologic history began with T1N1M0 oral cavity squamous cell carcinoma of the tongue diagnosed in March 2020, treated with right partial glossectomy and chemoradiotherapy. She subsequently experienced multiple recurrences requiring a right hemi-glossectomy and neck dissection in August 2022, followed by more extensive surgical resection with forearm skin grafting in January 2023. She initiated combination therapy with pembrolizumab, carboplatin, and paclitaxel in April 2024 and transitioned to pembrolizumab monotherapy in June 2024. The mucocutaneous findings developed approximately 5 months after starting pembrolizumab monotherapy. Pembrolizumab was held 5 weeks after symptom onset, and dupilumab therapy was initiated 1 week thereafter.

**Fig 1:**
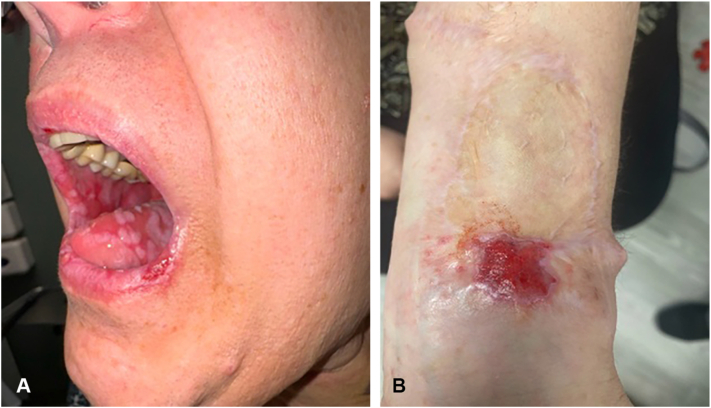
Erosive lichenoid eruption secondary to pembrolizumab. **A,***White plaques* with reticulated borders and areas of erosions on the lips. Widespread *white plaques* were present on the bilateral buccal and labial mucosa and dorsal tongue with interspersed erythematous erosions, creating a cobblestone appearance. **B,** Left volar wrist graft site with a well-demarcated, flat-topped ulcerated plaque demonstrating a *beefy red* granulating base and violaceous rim with fine *white scale*.

Oral examination revealed erosive white plaques involving the lips, bilateral buccal and labial mucosa, and dorsal tongue, creating an erythematous cobblestoned appearance (Fig 1, *A*). The left volar wrist graft site exhibited a well-demarcated, flat-topped ulcerated plaque with a beefy red granulating base and violaceous rim with fine white scale (Fig 1, *B*). No involvement of the genital mucosa or other cutaneous sites was identified. Biopsy of the wrist revealed focal ulceration of the epidermis with an adjacent lichenoid inflammatory infiltrate. Biopsy of the anterior tongue revealed erosion with a lichenoid interface dermatitis and mixed inflammatory infiltrate, with negative direct immunofluorescence. Initial treatment with viscous lidocaine, oral dexamethasone, and prophylactic fluconazole 200 mg weekly for 6 weeks yielded no improvement. Oral prednisone 60 mg daily provided partial symptomatic relief. Dupilumab was subsequently started with a 600 mg loading dose followed by 300 mg biweekly.

**Fig 2:**
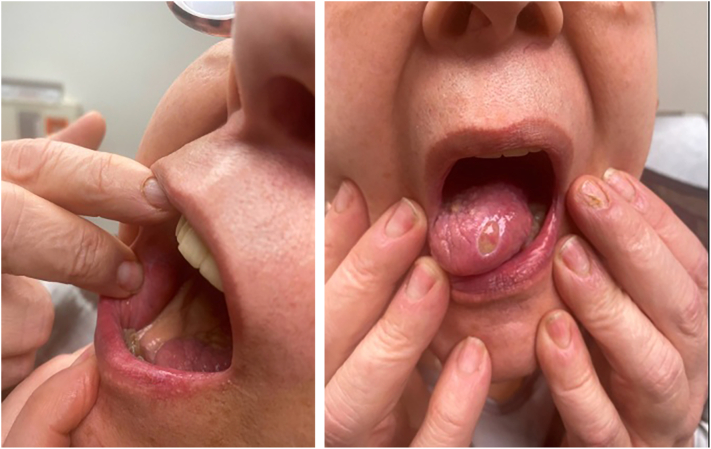
Resolving oral erosions with dupilumab and oral prednisone after 2 weeks of therapy.

Two weeks after initiating dupilumab, she showed marked improvement with no new mucocutaneous erosions (Fig 2). Prednisone was tapered down to 20 mg daily. Dupilumab was continued, and pembrolizumab was resumed 6 weeks later, with a mild recurrence of oral symptoms managed with continued dupilumab, topical corticosteroids, dexamethasone oral solution, and prednisone 10 mg daily. Approximately 3 months after starting dupilumab, prednisone was discontinued, and she remained well-controlled on dupilumab and topical steroids while continuing pembrolizumab.

## Discussion

Pembrolizumab-induced lichenoid reactions represent a rare but significant immune-related adverse event associated with ICIs, thought to result from T-cell activation triggered by ICI-mediated immune modulation.^7^

Most cases of ICI-induced LP involve cutaneous manifestations. Oral involvement is exceedingly rare, with only 2 previously reported cases of pembrolizumab-induced oral LP.^8^^,^^9^ In both cases, disease control was achieved with topical corticosteroids alone or in combination with systemic corticosteroids.

In contrast, our patient developed severe oral erosive LP involving the tongue, buccal mucosa, and lower lip after 5 months of pembrolizumab therapy. Her symptoms persisted despite treatment with high-dose systemic prednisone and topical corticosteroids. Pembrolizumab was temporarily held, and initiation of dupilumab led to significant improvement, permitting reintroduction of pembrolizumab with sustained control on dupilumab and low-dose prednisone.

Emerging literature supports the use of dupilumab in a range of ircAEs, particularly steroid-refractory or steroid-dependent cases.^4^^,^^5^^,^^10^^,^^11^ In a retrospective study of 39 patients treated with dupilumab for ircAEs, 87.2% showed clinical improvement, with complete resolution in 44.1% and partial but meaningful improvement in 55.9%.^4^ Eczematous and morbilliform drug eruptions (41% each) were most common, with spongiotic (46%), interface (21%), and perivascular (18%) dermatitis predominating histologically; lichenoid dermatitis occurred in 5%.^4^

Given dupilumab’s favorable safety profile and targeted inhibition of interleukin (IL)-4 and IL-13 signaling, it represents a promising steroid-sparing option for managing ircAEs. Our case highlights its successful use in oral erosive LP.

Paradoxical lichenoid eruptions—cutaneous, oral, and lichenoid-granulomatous—have been reported with dupilumab and have arisen de novo after therapy initiation rather than representing exacerbations of preexisting lichenoid dermatitis.^12^^,^^13^ LP is primarily driven by Th1 and IL-23/Th17 inflammatory pathways, with cytotoxic CD8^+^ T cells and IFN-γ mediating basal keratinocyte injury.^14^ By blocking IL-4 and IL-13 signaling, dupilumab suppresses Th2-mediated inflammation. This may disrupt immune homeostasis and promote Th1-skewed inflammation in susceptible individuals, resulting in paradoxical lichenoid eruptions.^12^^,^^13^ Due to these rare events, caution should be exercised when prescribing dupilumab to patients with preexisting lichenoid dermatoses, or Th1-predominant immune profiles.^12^^,^^13^ Monitoring for the development of paradoxical reactions is warranted when using dupilumab for ircAEs.

## Conclusion

Pembrolizumab-induced oral erosive LP is a rare but severe immune-related cutaneous adverse event. Dupilumab offers a promising treatment option for refractory cases.

## Conflicts of interest

None disclosed.
